# Daratumumab, carfilzomib, and pomalidomide for the treatment of POEMS syndrome: The Mayo Clinic Experience

**DOI:** 10.1038/s41408-023-00859-x

**Published:** 2023-05-31

**Authors:** I. Vaxman, S. K. Kumar, F. Buadi, M. Q. Lacy, D. Dingli, S. Hayman, T. Kourelis, R. Warsame, Y. Hwa, A. Fonder, M. Hobbs, E. Muchtar, N. Leung, P. Kapoor, R. Go, Y. Lin, W. Gonsalves, M. Siddiqui, R. A. Kyle, S. V. Rajkumar, M. A. Gertz, A. Dispenzieri

**Affiliations:** 1 Division of Hematology, Mayo Clinic, Rochester, MN USA; 2 Institute of Hematology, Davidoff Cancer Center, Rabin Medical Center, Petah- Tikvah, Israel; 3 Sackler Faculty of Medicine, Tel-Aviv University, Tel-Aviv, Israel

**Keywords:** Myeloma, Peripheral neuropathies

## Introduction

POEMS (Polyneuropathy, Organomegaly, Endocrinopathies, Monoclonal protein, Skin changes) syndrome is a rare paraneoplastic syndrome related to plasma cell disorders. The diagnosis is often delayed, and many patients are diagnosed and treated for chronic inflammatory demyelinating polyradiculopathy (CIDP) before the correct diagnosis of POEMS is made. Since the clinical manifestations of POEMS are debilitating with progressively deteriorating neurological symptoms, endocrine manifestations, and anasarca, early diagnosis and intervention are of paramount importance.

Therapy is aimed at eradicating the underlying plasma cells. Due to the rarity of this disease, randomized clinical trials to direct treatment selection are lacking apart from one randomized controlled trial assessing the use of thalidomide [1], and treatment decisions are based on case series and case reports [2–5]. Literature exists on the use of lenalidomide and bortezomib in the relapsed setting; however, data on the treatment of relapsed patients using daratumumab (D), carfilzomib (K), pomalidomide (P), and elotuzumab (E)-based therapies are scarce. Herein we describe our experience using these drugs for patients with relapsed POEMS syndrome.

## Material and methods

We identified all POEMS patients seen at Mayo Clinic Rochester, Minnesota using a prospectively maintained database. Of these patients, we identified all the patients that were treated with an agent-of-interest, defined as daratumumab, carfilzomib, pomalidomide, or elotuzumab. The study was approved by the Mayo Clinic Institutional Review Board. All patients authorized the use of their medical record data for research purposes. Data were extracted from the medical records.

The diagnosis of POEMS was according to consensus criteria [6]. Previously described POEMS response criteria were utilized [2, 7, 8], using laboratory (plasma VEGF), hematologic (M-spike, bone marrow plasma cells), radiologic (fluorodeoxyglucose (FDG) avidity on positron emission tomography (PET)), and clinical responses (Supplementary Table 1). An overall response was considered a partial response (PR) or better in any of the 4 domains without worsening in another domain. Individual domain responses are expressed as level of response with the domain as a subscript (V, H, P, and C). Time to next therapy (TTNT) was defined as the time from the institution of the regimen of interest to the next line of therapy.

Kaplan-Meier method was used for TTNT analysis, and all statistical tests were two-sided and P-values of < 0.05 were significant. Statistical analysis was carried out using JMP16 (SAS Institute, Cary, NC) statistical software.

## Results

### Characteristics at diagnosis

Of the 374 POEMS patients seen at Mayo Clinic, Rochester between January 1979 and May 2021, 16 patients received 24 regimens containing one of the agents-of-interest. All 16 patients were diagnosed between 2002 and 2018. Their median age at POEMS diagnosis was 57 years (range 39–79), and 15 patients (93%) were men (Supplementary Table 2), and none of the patients were treated due to a myeloma defining event other than a bone lesion.

First line therapies in our cohort included radiation (*N* = 5), cyclophosphamide (C) and methylprednisolone (*N* = 4), ixazomib+lenalidomide+dexamethasone (*N* = 2), lenalidomide and dexamethasone (Rd) (*N* = 3), bevacizumab (*N* = 1) and autologous stem cell transplantation (ASCT) without prior induction (*N* = 1). Twelve patients (75%) had prior ASCT.

### Treatment and response to agents-of-interest

**Fig. 1:**
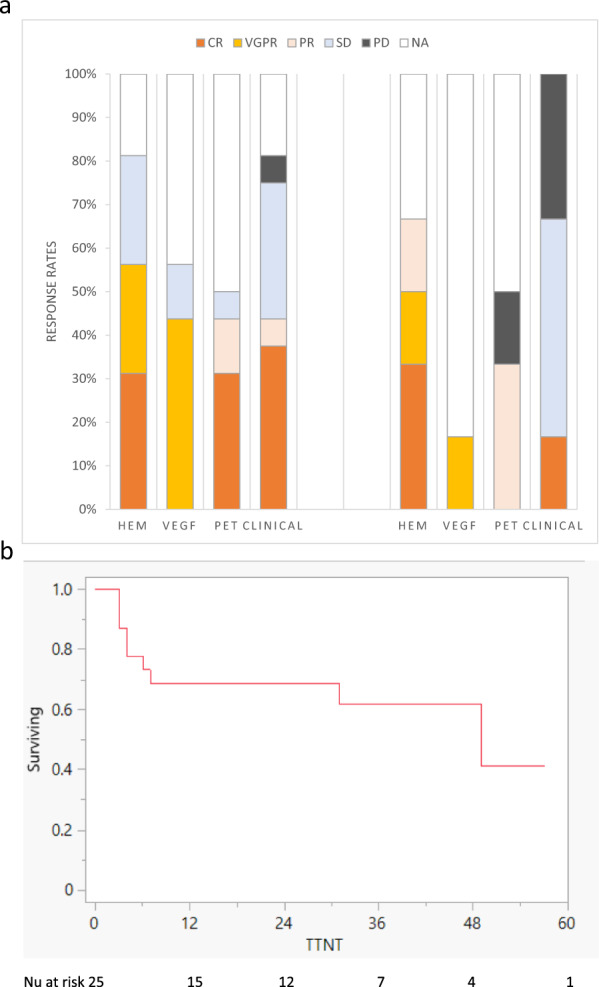
Response and survival. **a** Clinical, hematological, PET, and VEGF responses in POEMS patients treated with daratumumab-based therapies and carfilzomib-based therapies. **b** Time to next treatment.

Of these second line or later regimens, 5 (31%) were doublets including dexamethasone (d), 17 (71%) were triplets, and 2 (8%) were quadruplets. Median time from diagnosis to agent-of-interest was 50 months (IQR 23–122), and median lines of therapy was 2 (range 1–4). The median age was 63 years (IQR 51–70), and 4 (24%) were 70 years or older. The indications for therapy were clinical progression (*N* = 6), PET/CT progression only (*N* = 5), hematological progression only (*N* = 3), and VEGF progression (*N* = 2). The overall response rate was 62% (Fig. 1a; Table 1). At a median follow-up of 38 months (IQR 24–57) from initiation of the agent-of-interest, median TTNT was 48 months (95%CI 6-NR) (Fig. 1b). Fifteen patients (94%) are still alive. The one patient who died (Pt #16) received multiple lines of therapy, achieving a PR_H_ to KRd but no clinical response; he died off therapy due to acute respiratory failure.

**Table 1:** Response to novel agents of interest.

Pt ID	Regimen	Prior lines Rx, *N*	Dx to NAOI Rx, months	Duration of Rx, months	TTNT, months	Hematologic	VEGF	PET	Clinical
**5**	**Dd**	**2**^**a,b**^	**35**	**2**	**4**	**SD**	**NA**	**NA**	**PD**
10	Dd	3^b^	129	42^c^		VGPR	NA	PR	NA
14	DRd	4	125	12^c^		CR	CR	NA	NA
8	DRd	1^b^	100	13^c^		NA	CR	CR	NA
6	DRd	3^a,b^	47	15		CR	CR	NE	CR
4	DRd	1^a,b^	16	31^c^		NA	NA	CR	CR
11	DRd	1^b^	138	37		CR	CR	CR	PR
2	DRd	1	27	56^c^		VGPR	CR	CR	CR
**16**	**DPd**	**5**^**a**^	**46**	**1**		**SD**	**SD**	**SD**	**SD**
13	DPd	2^b^	24	30	31	CR	NA	CR	CR
9	DPd	3^a,b^	135	32^c^		VGPR	NA	PR	SD
7	DPd	3^b^	53	46		CR	CR	NA	SD
3	DCd	1^b^	19	3		NA	CR	NA	CR
**16**	**DCd**	**3**^**a**^	**36**	**4**	**7**	**SD**	**NA**	**NA**	**SD**
1	DVCd	1^a^	22	3	4	VGPR	NA	NA	CR
**1**	**DPVd**	**2**^**a**^	**26**	**16**		**SD**	**SD**	**NA**	**SD**
9	Kd	2^a,b^	86	28	49	VGPR	NA	PR	SD
12	Kd	4^a,b^	141	57^c^		CR	CR	NA	CR
**13**	**KRd**	**1**^**b**^	**22**	**2**	**3**	**NA**	**NA**	**PD**	**PD**
**16**	**KRd**	**2**^**a**^	**31**	**5**	**6**	**PR**	**NA**	**NA**	**PD**
15	KRd	1	165	16^c^		NA	NA	PR	SD
5	KPd	3^a,b^	38	36^c^		CR	NA	NA	SD
**12**	**Pd**	**2**^**a,b**^	**116**	**2**	**3**	**PD**	**PD**	**NA**	**PD**
**16**	**ERd**	**4**^**a**^	**43**	**2**	**3**	**SD**	**SD**	**SD**	**SD**

Bold rows refer to non-responding regimen/patient.

*C* cyclophosphamide, *CR* complete response, *D* daratumumab, *d* dexamethasone, *Dx* diagnosis, *E* elotuzomab, *K* carfilzomib, *mo* months, *NA* not assessed, *P* pomalidomide, *PD* progressive disease, *PR* partial response, *Pt* patient, *R* lenalidomide, *Rx* therapy, *SD* stable disease, *TTNT* time to next therapy, *V* bortezomib, *VEGF* vascular endothelial growth factor, *VGPR* very good partial response.

^a^Patients who received prior lenalidomide.

^b^Patients who had prior ASCT.

^c^Therapy is ongoing.

The daratumumab-based therapies included: 2 with dexamethasone alone; 10 as part of an IMID (immune modulator drug)-steroid-triplet (lenalidomide, 6; pomalidomide, 4); 2 in combination with cyclophosphamide; and 2 as part of a quadruplet (DVCd and DPVd). Of the 16 daratumumab-based therapies, there were 12 (75%) responses (Fig. 1a): CR/VGPR_H_, 9 (56%); CR_V_,7 (44%); and CR_P_, 5 patients (31%). One of the Dd patients had progressive disease, and the other responded (VGPR_H_, PR_P_). Among the D-IMID-triplet regimens, there were 9 (75%) responses. Of the 3 daratumumab-triplet/quadruplet regimens without response, 2 were administered to the refractory patient who died; the third occurred in a patient who had responded to DVCd but was transitioned to DPVd to avoid long-term alkylator use; no further response was seen, but patient remained on regimen for 16 months. The TTNT in the Daratumumab-based regimens yielded was not reached.

Of the 6 patients treated with carfilzomib-based therapies, 5 patients (83%) responded to therapy: 3 patients achieved CR/VGPR_H_, 1 achieved PR_H_, and 1 achieved a clinical response (Fig. 1a). The 2 Kd-treated patients responded. Of the 4 patients treated with carfilzomib, IMID, and steroid triplet (3 KRd and 1KPd), 3 responded, with a median TTNT of 49 months (95%CI 3-NR).

One patient received elotuzumab with lenalidomide and dexamethasone (ERd) and did not achieve either clinical or a hematological response; therapy was discontinued after 2 months. Of note, this was Pt#16, who also did not respond to DCd or DPd.

None of the patients discontinued therapy due to adverse events, and no death case occurred while on therapy. Adverse events are presented in Supplementary Table 3 and included 7 events of hospitalization due to pneumonia (4 in daratumumab-based therapies and 3 on carfilzomib-based therapies) and 4 patients hospitalized due to volume overload. Three patients experienced infusion-related reactions (IRR) to the first dose of IV daratumumab.

## Discussion

This is the largest systematic report on the outcomes of patients with relapsed or refractory POEMS who were treated with daratumumab, carfilzomib, pomalidomide, or elotuzumab. Therapy with these drugs was effective, responses were durable, and therapy was safe. Twelve daratumumab-based regimens (75%) and five carfilzomib-based therapies (83%) resulted in responses.

More than half of the daratumumab-based treatments resulted in a VGPR_H_ or better even though 12 of the 16 were treated with either lenalidomide, bortezomib, or thalidomide as first line. We can say little about the value of the doublet Dd since only 2 patients received this combination (50% response rate); however, of the 10 patients treated with a D-IMiD-d combination, median duration of therapy exceeded 2.5 years, which approaches the PFS of 3.7-year observed with DRd for patients with multiple myeloma (MM) and exceeds the PFS of 1-year seen with DPd for patients with MM [9, 10]. To date, there are only 2 case reports of daratumumab use in POEMS syndrome: one relapsed patient with POEMS syndrome [11] and two patients in the upfront setting: [5] all three patients received DRd, and all responded very well in terms of neurological response, VEGF response, and hematological response.

Because bortezomib can cause neuropathy, it is of interest to understand the utility of carfilzomib in a POEMS population. Both patients treated with Kd responded with TTNT of more than 4 years. Though small numbers, these results are comparable to what is seen in MM (response rate 73–84% and PFS of 1.3 to 2.4 years) [5]. Two of the 4 patients treated with K-IMID triplets responded, but their TTNT was quite variable, ranging from 2 months to 3 + years.

Four of the seven pomalidomide-treated patients responded; notably, the one patient receiving a doublet did not respond, but 3 of the 4 DPd patients did respond as did the one KPd-treated patient. Median duration of treatment for pomalidomide-treated patients was 2.5 years. These results compare favorably to the reported experience in the relapsed setting using Rd in patients with POEMS syndrome (from 48 to 95% [12–14]) and using DPd or Pd in MM patients (46 to 69% response rate and PFS of approximately 0.6 to 1.0 year) [10].

This study has several limitations. Because this is a retrospective report from a referral institution, follow-up was not standardized, and missing data precluded our ability to report on hematologic, VEGF, and PET-CT responses and progressions at designated time-points for all patients, and TTNT was used as a surrogate for PFS. Hematologic response and progression are often not measurable given the low “tumor burden” of patients with POEMS syndrome; therefore, both individual system responses and composite responses were reported.

Despite these limitations, this work is important since it is the largest report on the use of daratumumab, carfilzomib, pomalidomide, and elotuzumab in patients with POEMS syndrome. Responses were common and durable, and no novel toxicities were appreciated. Due to small numbers of patients, we could not surmise which therapies are best, but it is likely that triplets are better than doublets, as is the case for MM. Future studies are needed to clarify the optimal combinations and sequences of novel agents.

## Supplementary information

supplementary tables

## Data Availability

The datasets generated and analyzed during the current study are not publicly available but are available from the corresponding author on reasonable request.
