# Supplementary material for: Daratumumab, carfilzomib, and pomalidomide for the treatment of POEMS syndrome: The Mayo Clinic Experience
Source: Blood Cancer J. 2023 May 31;13(1):91. doi: 10.1038/s41408-023-00859-x (PMC10229532; doi:10.1038/s41408-023-00859-x)
Supplement: Supplementary file 1 — supplementary tables [file 41408_2023_859_MOESM1_ESM.docx]

Supplementary Table 1- Baseline Characteristics of Study Population (N=16)

| Characteristics | N=16 |
| --- | --- |
| Male, N (%) | 15 (94) |
| Age at diagnosis, years, median (range) | 57 (39-79) |
| Seen at Mayo 90 days of diagnosis, N (%) | 11 (69) |
| Polyneuropathy, N (%) | 16 (100) |
| Hepatomegaly or splenomegaly, N (%) | 5 (30) |
| Splenomegaly, N (%) | 7 (44) |
| Castleman variants, N (%) | 1 (6) |
| Endocrinopathy, N (%) | 15 (94) |
| Skin changes, N (%) | 10 (63) |
| Extravascular volume overload (edema, pleural effusion, ascites), N (%) | 15 (94) |
| Papilledema, N (%) | 4 (25) |
| Osteosclerotic lesions, N (%) | 13 (81) |
| ECOG, N (%)  1 / 2  3 / 4 | 9 (56) / 4 (25)  1 (6) / 2 (13) |
| Plasma VEGF pg/mL, median (IQR) | 584 (335-1053) |
| Serum albumin, g/dL, median (IQR) | 2.9 (2.5-3.4) |
| Hemoglobin, g/dL, median (IQR) | 15.5 (13.5-16.1) |
| Platelets, median (IQR) | 443 (317-667) |
| Lambda mg/dL, n (%) | 16 (100) |
| IgA mg/dL, N (%) | 10 (63) |
| IgG, N (%) | 6 (37) |
| Bone marrow plasma cells, median (IQR) | 6 (1-10) |
| Bone marrow plasma cells over 10%, n (%) | 6 (38) |
| First line treatment, N (%)  ASCT  Radiation  Rd  IRd  Cyclophosphamide+ solumedrol  Avastin | 12 (75)  5 (31)  3 (19)  2 (13)  4 (25)  1 (6) |
| Indication for NAOI  Clinical relapse  Rising VEGF  PET positivity  Rising monoclonal protein | 6 (37)  2 (13)  5 (31)  3 (19) |

ASCT=autologous stem cell transplantation; IRD=ixazomib, lenalidomide and dexamethasone; N=number; NAOI, novel agent of interest; RD=lenalidomide and dexamethasone; VEGF=vascular endothelial growth factor

Supplementary table 2. Definition of response

| Response * | | Complete response | Very good partial response | Partial response | No response | Progressive disease |
| --- | --- | --- | --- | --- | --- | --- |
| Hematologic | | CR_H-_ negative immunofixation of the serum and no clonal plasma cells in the marrow | VGPR_H_-90% reduction in M-protein or immunofixation positive (given that M-protein was at least 0.5 g/dl at baseline | PR_H_-50% reduction in M-protein or immunofixation positive as long as baseline M-protein was at least 1.0 g/dl | NR_H_- not meeting criteria for CR_H_, VGPR_H_ or PR_H_ | hematologic progression- an increase in the M-component by 25% from the lowest value |
| PET | CR_P_- an initial FDG avidity on a baseline PET scan that disappears after treatment | |  | PR_P_- an initial FDG avidity that was 50% improved after treatment | NR_P_- all cases that had initial FDG avidity but did not meet the criteria for CRP or PRP | PET progression -increase in size or FDG avidity of existing plasmacytomas on PET scan |
| VEGF | | CR_V_- normalized VEGF |  | PR_V_- VEGF improved by at least 50% (assuming baseline was 200 pg/ml) | NRv- not meeting either CR or PR. | VEGF progression, persistent increase in plasma VEGF level >200 pg/ml on at least 2 occasions |

* Clinical responses to therapy were reported by the treating physician and included complete clinical response (CR_C_), partial clinical response (PR_C_), stable disease (SD_C_), and progressive disease (PD_C_).

Supplementary table 3- Adverse events to novel therapies of interest

|  | Infections | Hospitalizations | Hematological toxicities | IRR | GI toxicities |
| --- | --- | --- | --- | --- | --- |
| Dara-dex; DRd; Dara-VCd; Dara-PVd | Influenza A X1, pneumonia X1 | Hospitalized due to volume overload X3 | Pancytopenia X1 | IRRX3 (one Grade 3) | diarrheaX1, NauseaX1 |
| Kyprolis-dexa; KRd | Hospitalized due to pneumonia X4 | Hospitalized due to volume overload X1 | Thrombocytopenia X1 |  | Hospitalized due to diarrhea X1, nausea so lenalidomide discontinued |
| DPD | Hospitalized due to pneumonia and sepsis X1 |  | Neutropenia G3 X1 |  |  |

DPD= daratumumab, pomalidomide, and dexamethasone; IRR=infusion related disorders; G3=grade 3; dex=dexamethasone; Dara-VCd=daratumumab, bortezomib, dexamethasone; Dara-PVd=daratumumab, pomalidomide, dexamethasone; DRD-= daratumumab, lenalidomide, dexamethasone; KRd=Carfilzomib, lenalidomide, dexamethasone
